# Investigation and Management of Apparently Sporadic Central Nervous System Haemangioblastoma for Evidence of Von Hippel–Lindau Disease

**DOI:** 10.3390/genes12091414

**Published:** 2021-09-15

**Authors:** Hugh Furness, Louay Salfity, Johanna Devereux, Dorothy Halliday, Helen Hanson, Deborah M. Ruddy, UK VHL Study Group, Neha Shah, George Sultana, Emma R. Woodward, Richard N. Sandford, Katie M. Snape, Eamonn R. Maher

**Affiliations:** 1 Department of Clinical Genetics, St George’s Healthcare NHS Trust, London SW7 0RE, UK; hughfurness@hotmail.com (H.F.); louaysalfity@gmail.com (L.S.); helen.Hanson@stgeorges.nhs.uk (H.H.); ksnape@sgul.ac.uk (K.M.S.); 2 Department of Clinical Genetics, Guy’s Hospital, London SE1 9RT, UK; johanna.devereux@nhs.net (J.D.); Deborah.Ruddy@gstt.nhs.uk (D.M.R.); 3 Oxford Centre for Genomic Medicine, Oxford University Hospitals NHS Foundation Trust, Oxford OX3 7HE, UK; dorothyhalliday@nhs.net; 4 Department of Medical Genetics, University of Cambridge, Cambridge CB2 0QQ, UK; 5 Cambridge University Hospitals NHS Foundation Trust, Cambridge CB2 0QQ, UK; ns309@medschl.cam.ac.uk (N.S.); georgesultana17@gmail.com (G.S.); rns13@medschl.cam.ac.uk (R.N.S.); 6 Manchester Centre for Genomic Medicine, St Mary’s Hospital, Manchester University Hospitals NHS Foundation Trust, Manchester M13 9WL and Division of Evolution and Genomic Sciences, School of Biological Sciences, Faculty of Biology, Medicine and Health, University of Manchester, Manchester Academic Health Science Centre, Manchester M13 9PL, UK; Emma.Woodward@mft.nhs.uk

**Keywords:** haemangioblastoma, renal cell carcinoma, genetics, VHL

## Abstract

Haemangioblastomas are rare, highly vascularised tumours that typically occur in the cerebellum, brain stem and spinal cord. Up to a third of individuals with a haemangioblastoma will have von Hippel–Lindau (VHL) disease. Individuals with haemangioblastoma and underlying VHL disease present, on average, at a younger age and frequently have a personal or family history of VHL disease-related tumours (e.g., retinal or central nervous system (CNS) haemangioblastomas, renal cell carcinoma, phaeochromocytoma). However, a subset present an apparently sporadic haemangioblastoma without other features of VHL disease. To detect such individuals, it has been recommended that genetic testing and clinical/radiological assessment for VHL disease should be offered to patients with a haemangioblastoma. To assess “real-world” clinical practice, we undertook a national survey of clinical genetics centres. All participating centres responded that they would offer genetic testing and a comprehensive assessment (ophthalmological examination and CNS and abdominal imaging) to a patient presenting with a CNS haemangioblastoma. However, for individuals who tested negative, there was variability in practice with regard to the need for continued follow-up. We then reviewed the results of follow-up surveillance in 91 such individuals seen at four centres. The risk of developing a potential VHL-related tumour (haemangioblastoma or RCC) was estimated at 10.8% at 10 years follow-up. The risks of developing a recurrent haemangioblastoma were higher in those who presented <40 years of age. In the light of these and previous findings, we propose an age-stratified protocol for surveillance of VHL-related tumours in individuals with apparently isolated haemangioblastoma.

## 1. Introduction

Haemangioblastomas are rare, benign vascular tumours, most commonly located in the cerebellum, brain stem and spinal cord. They account for 1–2% of all intracranial neoplasms and up to 12% of posterior fossa tumours [1]. The majority occur in the cerebellum (76%) and much less frequently in the cerebral hemispheres (9%), spinal cord (7%) and brainstem (5%) [1].

The incidence of intracranial haemangioblastomas is reported as ~1.5 per million person-years, and haemangioblastomas typically occur between the second and eighth decades and may be isolated or multiple [2]. Haemangioblastomas are highly vascular tumours composed predominantly of vascular and stromal cells, with the latter representing the neoplastic component of the tumour [3]. In most cases (~70%), haemangioblastomas are sporadic, but about 30% are associated with von Hippel–Lindau (VHL) disease, an autosomal dominantly inherited disorder characterised by the development of retinal and central nervous system haemangioblastomas, clear cell renal cell carcinoma (cRCC), phaeochromocytoma/paraganglioma (PPGL), pancreatic neuroendocrine tumours (PNET), endolymphatic sac tumours (ELST) and visceral cysts [4,5,6]. Around 20% of individuals with VHL disease do not have a prior family history of the disease, which results from *de novo* mutations in the *VHL* gene [7]. Individuals with VHL disease have, on average, an earlier age at diagnosis of haemangioblastomas than sporadic cases (e.g., 29 years versus 48 years) [4] and are predisposed to develop multiple haemangioblastomas [8]. However, VHL disease exhibits age-related penetrance and variable expression; therefore, a diagnosis of VHL disease should be considered in all individuals who present with a haemangioblastoma irrespective of family history [9].

A diagnosis of VHL disease can be made by clinical diagnostic criteria or by the detection of a germline pathogenic variant in the *VHL* gene [5,10]. Clinical diagnostic criteria require the presence of a single VHL-related neoplasm (e.g., haemangioblastoma, cRCC, PPGL, PNET, ELST) in an individual with a confirmed family history of VHL disease or, if there is no family history, two haemangioblastomas or a haemangioblastoma and a second VHL-related tumour [5,8,11]. Therefore, individuals with VHL disease resulting from a *de novo* mutation who present with a single CNS haemangioblastoma can only fulfil clinical diagnostic criteria when developing a second tumour. As such, delayed diagnosis may lead to avoidable mortality and morbidity, and it was reported previously that 4% of patients with an apparently sporadic CNS haemangioblastoma and no clinical evidence of VHL disease will have a detectable *VHL* gene mutation [12]. Within the UK, it is now routine to offer genetic testing to individuals who present with a haemangioblastoma under the age of 60 years, and it also has been suggested that testing should be considered in all cases [13]. Individuals in whom a molecular diagnosis of VHL disease is made can then be entered into a VHL disease surveillance programme and their at-risk relatives offered pre-symptomatic genetic testing [12,13]. For individuals with an apparently sporadic haemangioblastoma and a negative *VHL* genetic test, the risk of cryptic VHL disease is significantly diminished; however, the sensitivity of *VHL* gene testing is ~95%, and false negative tests may occur through mosaicism or the location of pathogenic variants in intronic regions [14,15,16]. Individuals with a haemangioblastoma at a young age or potential (but inconclusive) evidence of VHL disease (e.g., multiple renal cysts) are likely to cause most concern about undiagnosed VHL disease, but there is little information on how such individuals should be managed. To facilitate the development of evidence-based clinical pathways for the follow-up of patients with apparently sporadic CNS haemangioblastomas, we audited the current practice in UK clinical genetics centres and the detailed results of follow-up surveillance in 91 individuals from four genetics centres.

## 2. Methods

### 2.1. Audit of Current Practice of Follow-Up for Sporadic CNS Haemangioblastomas

A national audit to establish current clinical practice for management of individuals with (or suspected of having) VHL disease was conducted through 21 clinical genetics centres in the United Kingdom (UK). In addition to answering questions about patients attending their centre with a clinical or molecular diagnosis of VHL disease, each centre was asked, “Does your service routinely see patients with an apparently isolated CNS haemangioblastoma?” For those centres that answered “yes”, there were five supplementary questions: (a) “Are there any age criteria?”, and for patients with an isolated CNS haemangioblastoma and a negative *VHL* gene test during 2012–2017: (b) “How many have been screened?”; (c) “Are your screening criteria the same as those for patients with VHL disease?”; (d) “Do you offer screening to other family members and, if so, who?”; and (e) “At what age do you discontinue screening if no other features of VHL disease are found?”

### 2.2. Audit of Surveillance Results in Individuals with Sporadic CNS Haemangioblastoma and Negative VHL Gene Testing

A retrospective study was conducted on 91 patients with sporadic CNS haemangioblastomas and no evidence of germline *VHL* mutation on genetic testing. Data collection (location of haemangioblastoma, age at diagnosis, genetic testing results and findings from post-surgical surveillance) took place across four centres in the United Kingdom: Cambridge University Hospitals NHS Trust (*n* = 46 patients), Oxford University Hospitals NHS Trust (*n* = 30), and St George’s and Guy’s and St Thomas’s Hospitals NHS Trusts, London (*n* = 15). Kaplan–Meier analysis was performed using the MedCalc software package to estimate risks of potential VHL-related findings during the follow-up period. Approval for a clinical audit/service evaluation was obtained from each of the NHS Trusts.

## 3. Results

### 3.1. Audit of Current Practice of Follow-Up for Sporadic CNS Haemangioblastomas in UK Clinical Genetic Centres

Information on current practice for management of individuals with apparently sporadic haemangioblastomas was received from 21 Clinical Genetics Centres. Over the study year period (2012–2017) that was audited, the total number of individuals seen was 153 (mean 9.6 per centre, range 1 to 35). All of the centres responded that they would offer a comprehensive (ophthalmological examination and CNS and abdominal imaging) and genetic testing to a patient presenting with a CNS haemangioblastoma. However, there was variability in practice with regard to the continued follow-up. Five centres responded that they did not have a formal policy or did not specify one, and two centres performed only a single screen with no subsequent follow-up (if results were normal). However, most centres (*n* = 14) would continue to offer surveillance to an individual with an apparently isolated CNS haemangioblastoma and negative genetic testing (up to age 40 years in 4 centres, 50 years in 8 and 60 years in 2 centres; some centres reduce the frequency of surveillance in older age groups). None of the clinical genetics centres had a policy of screening first-degree relatives of the probands.

### 3.2. Audit of Surveillance Results in 91 Individuals with Sporadic CNS Haemangioblastoma and Negative VHL Gene Testing

**Figure 1:**
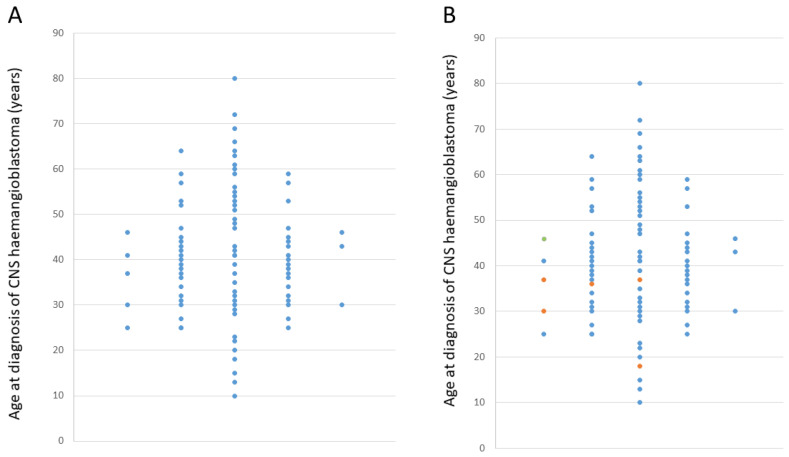
(**A**) Age-at-diagnosis distribution of sporadic haemangioblastoma patients who underwent post diagnosis surveillance. (**B**) Age-at-diagnosis distribution of sporadic haemangioblastoma patients who underwent post diagnosis surveillance with cases who developed a recurrent haemangioblastoma (orange) or renal cell carcinoma (green) (see details in Table 1). Mean age of diagnosis was 41 years (standard deviation 13.1 years; range 10–80 years).

*Patient characteristics:* 78 patients (85.7%) had cerebellar haemangioblastomas, 7 (7.7%) had spinal haemangioblastomas and 3 (3.3%) had brainstem haemangioblastomas (for 3 patients, the precise location was not available). Mean age of diagnosis was 41 years (standard deviation 13.1 years; range 10–80 years), and the age distribution is shown in Figure 1A.

*Surveillance-related findings:* All individuals underwent routine clinical and imaging tests to identify subclinical features of VHL disease (e.g., ophthalmology review, CNS imaging with CT/MRI and abdominal imaging (ultrasound or MRI). Clinical findings and the age at which haemangioblastomas were detected are detailed in Table 1 and Figure 1B. The most common findings were a further haemangioblastoma (either recurrence at original site or at a new site, two developing recurrence within the first year of follow-up, two at 5–6 years of follow-up and one developing a recurrence at 40 years after their initial diagnosis) and renal cysts, which occurred in 10 patients (a single cyst in six patients and multiple renal cysts in four patients). One patient developed a 4 cm renal cell carcinoma within a year of follow-up. Findings that were not considered as suggestive of VHL disease included a meningioma (*n* = 1 patient), liver haemangioma (*n* = 2), benign adrenal lesions (*n* = 2) and pancreatic lesions (*n* = 2).

**Figure 2:**
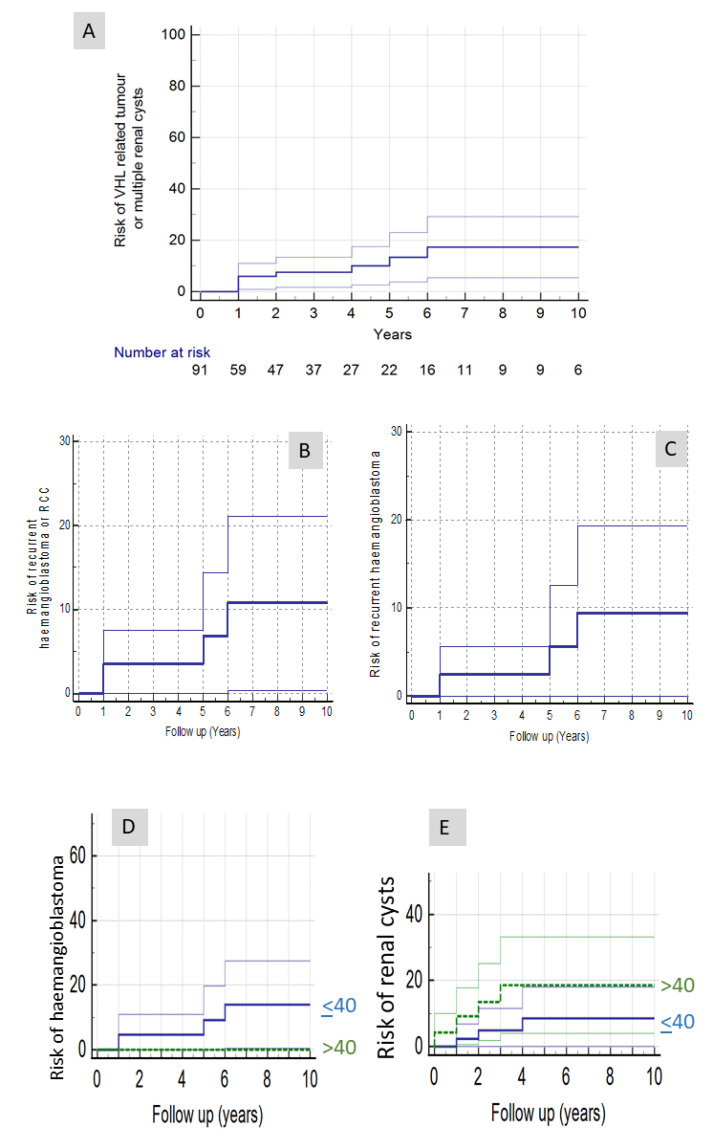
(**A**) Kaplan–Meier analysis of risk of developing a potential VHL-related feature (recurrent haemangioblastoma, renal cell carcinoma or multiple renal cysts) during surveillance in *VHL* mutation-negative haemangioblastoma patients. (**B**) Kaplan–Meier analysis of risk of developing a recurrent haemangioblastoma or renal cell carcinoma during surveillance in *VHL* mutation-negative haemangioblastoma patients. (**C**) Kaplan–Meier analysis of risk of developing a recurrent haemangioblastoma during surveillance in VHL mutation-negative haemangioblastoma patients. (**D**) Comparison of risks of developing a further haemangioblastoma in individuals whose haemangioblastoma was diagnosed <40 years (Group 1) and those aged >40 years (Group 2). (**E**) Comparison of risks of developing one or more renal cysts during follow-up in individuals whose haemangioblastoma was diagnosed <40 years (Group 1) and those aged >40 years (Group 2).

The overall risk of developing a potential VHL-related feature (haemangioblastoma, RCC or multiple renal cysts) was 17.3% at 10 years follow-up (Figure 2A), and the risk of developing a potential VHL-related tumour (haemangioblastoma or RCC) was 10.8% at 10 years follow-up (Figure 2B). The risk of developing a further haemangioblastoma was 9.4% at 10 years of follow-up (Figure 2C). In view of the younger mean age at diagnosis of a CNS haemangioblastoma in patients with VHL disease than in those with a sporadic haemangioblastoma, we estimated the risks for developing a further CNS haemangioblastoma separately for those whose initial haemangioblastoma was diagnosed <40 years (*n* = 43) and those aged >40 years (*n* = 48). This revealed 10-year risks of a further haemangioblastoma of 14% and 0%, respectively (log rank testing over whole follow-up period χ^2^ = 2.1761 *p* = 0.14) with all five further haemangioblastomas occurring in younger onset individuals (Figure 2D).

The risk of developing one or more renal cysts was 13.3% at 10 years of follow-up. The risks were then compared for those whose initial haemangioblastoma was diagnosed <40 years (*n* = 43) and those aged >40 years (*n* = 48). This revealed 10-year risks of a developing one or more renal cysts of 8.6% and 13.3%, respectively (log rank testing over whole follow-up period χ^2^ = 4.1985 *p* = 0.041), with three younger onset individuals developing renal cysts and seven in the older group (Figure 2E).

## 4. Discussion

Clinical presentation with an apparently sporadic haemangioblastoma is a well-recognised feature of VHL disease [8,9]. Accordingly, all UK clinical genetics centres that participated in the national audit reported that they would offer a comprehensive “one-off VHL screen” (comprising ophthalmological review, CNS and abdominal imaging and genetic testing for VHL disease) to patients at any age who presented with a CNS haemangioblastoma. Previously, we found that in a cohort of 188 individuals presenting with a single isolated haemangioblastoma, seven (3.7%) patients had a germline VHL variant [12]. Reassessing these variants in the light of ACMG/AMP variant interpretation criteria, four would be considered to have pathogenic variants, one a variant of uncertain significance, and two had a heterozygous missense variant (NM_000551.4(VHL):c.598C > T (p.Arg200Trp)) that is pathogenic for Chuvash polycythaemia in the homozygous state but is not now considered to be a risk factor for VHL-related tumours [17,18]. In addition, in the cohort reported by Woodward et al. (2007), 10 of 181 individuals without a germline VHL variant went on to develop possible evidence of VHL disease (nine developed further haemangioblastomas and one developed an RCC). For these 10 individuals, the average age at diagnosis of the initial haemangioblastoma presentation was 29.3 years (range 11 to 41 years), and the mean time to the second event was 12.3 years. By Kaplan–Meier analysis, it was estimated that there was a 5% risk of developing a VHL-type tumour after 10 years of follow-up. In the current cohort, the 10-year risks of a further haemangioblastoma were 14% diagnosed <40 years and 0% in those who presented with their initial haemangioblastoma after age 40 years, and so the current study results are broadly similar to those of Woodward et al. (2007). Similarly, in the current study, the 10-year risk of developing renal cysts was higher in individuals who were aged >40 years at haemangioblastoma diagnosis, and in the previous cohort, the actuarial risk of developing a simple renal cyst post haemangioblastoma diagnosis was 6.6% at 10 years, and the mean age at diagnosis of haemangioblastoma in those cases who developed a renal cyst was 49 years [12].

**Figure 3:**
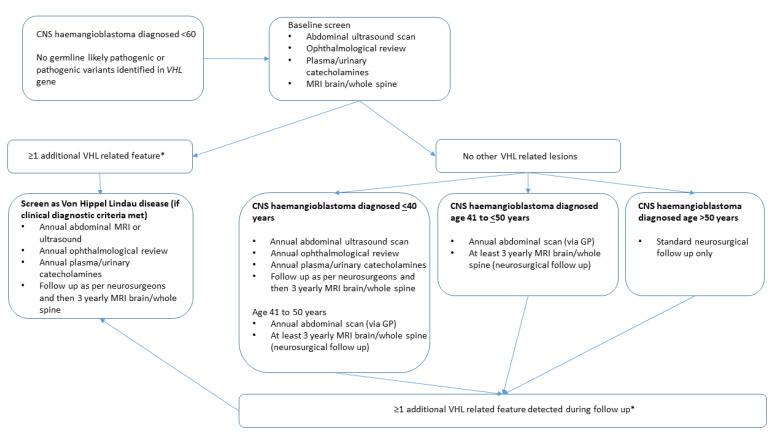
Proposed protocol for investigation and surveillance of individuals presenting with a central nervous system haemangioblastoma and no personal or family history of von Hippel–Lindau disease and negative genetic testing. * The interpretation of potential VHL-related features should be performed in the context of the overall clinical picture (e.g., the presence of renal cysts in an older individual (e.g., 70 years) may be unrelated to VHL disease, whereas multiple renal cysts before age 30 years would be a more significant finding).

Individuals who subsequently developed a VHL-related tumour might have had a false negative genetic test or be phenocopies with respect to VHL disease through recurrence of the original haemangioblastoma or had a coincidental occurrence of another VHL-type tumour. False negative genetic testing can result from variants that are not detected by standard testing techniques (e.g., intronic variants and rearrangements) and by mosaic mutations. In the past decade, in most centres, standard *VHL* gene testing strategies have progressed from Southern analysis and Sanger sequencing to MLPA and next-generation sequencing-based approaches [14,15,16]. It might be predicted that this shift in testing methodology would result in better detection of mosaic cases, and, therefore, recent patients who presented with an isolated sporadic haemangioblastoma would have a lower risk of developing further VHL-related tumours than the cohort reported previously [12]. That being said, we found no evidence of such a reduction. Therefore, it appears that the previous recommendation of surveillance in higher-risk sporadic haemangioblastoma patients, even if they have negative molecular testing, is still valid [12]. We found that the policy of the clinical genetics centres surveyed was to perform baseline screening for subclinical evidence of VHL disease, but there was intercentre variation in their policy towards continuing surveillance. Whilst all the centres recognised that false negative VHL gene testing was more likely in patients with early onset haemangioblastomas than those at an older age, both because the average age at diagnosis is younger in VHL disease than in sporadic cases and because other manifestations of VHL disease (e.g., RCC and visceral cysts) will be more penetrant in older patients, the age at which follow-up continued varied from age <40 to 60 years. Previously, Woodward et al. (2007) reported that the mean time to a second VHL-related tumour was 12.3 (maximum 22) years. Though some centres discharged patients from follow-up at age 40 years, we found in the current study that three patients developed a further haemangioblastoma after this age (two between 40 and 50 years), and one patient developed an RCC at age 47 years (each of the five patients who developed a recurrent haemangioblastoma after age 40 years had developed their primary haemangioblastoma aged <40 years (see Table 1)). We propose that if a common policy for follow-up of patients with *VHL* mutation-negative isolated CNS haemangioblastoma is to be adopted, then current evidence would suggest that patients should be kept under surveillance until age 50 years (though a small number of individuals would present with a second tumour at a later age). Individuals presenting with a haemangioblastoma aged >50 years would be offered comprehensive assessment for subclinical and molecular evidence of VHL disease and should then be discharged if their results are negative. Further details of such a protocol for managing the follow-up of individuals with apparently sporadic haemangioblastoma is provided in Figure 3. Individuals are stratified according to their age at diagnosis of a haemangioblastoma with younger onset cases followed up until age 50 years and individuals diagnosed with a sporadic CNS haemangioblastoma >50 years not offered follow-up for the development of VHL disease-related complications (just standard neurosurgical follow-up) if genetic testing is negative and baseline screening investigations show no evidence of VHL disease. Among younger onset cases, more intense surveillance is offered until age 40 years, whereas from age 41 to 50 years, standard neurosurgical follow-up and annual abdominal ultrasonography (organised locally via their general practitioner) are suggested with a low threshold for re-referral. It is hoped that this protocol would enable the detection of most later-onset VHL-type manifestations but would not involve patients having to undergo extensive annual investigations. The detection of potential VHL-related features during follow-up would, however, require re-evaluation of the possibility of underlying VHL disease. Our proposed protocol reflects the current unavailability of funding for *VHL* testing in patients who present with a haemangioblastoma after age 60 years. As can be seen in Figure 1, in our retrospective study, only a small number of patients aged >60 years had been tested (~11%). After the age of 60 years, other clinical features of VHL disease are likely to be present, but further data on the utility of genetic testing in older patients with a central nervous system haemangioblastoma would inform clinical management.

Stromal cells within VHL-related and VHL disease-independent haemangioblastomas demonstrate biallelic *VHL* inactivation, but the frequency of other genetic events might differ [19,20,21]. Longer term, the identification of tumour biomarkers that might distinguish between VHL-related and VHL disease-independent haemangioblastomas [22] could enable better stratification of sporadic haemangioblastoma patients and a more personalized approach to continuing surveillance.

## 5. Conclusions

Based on our findings of VHL-related features developing during follow up in patients with apparently sporadic CNS haemangioblastoma we suggest a follow up protocol described in Figure 3.

## Figures and Tables

**Table 1:** Patients who developed potential VHL-related findings after diagnosis of their CNS haemangioblastoma. The total follow-up in years after diagnosis and the age at which the features developed is displayed.

Patient	Age at Diagnosis of Haemangioblastoma	Total Follow-Up Time (in Years)	Clinical Feature	Age at Detection
HAB1	47	3	Solitary renal cyst	48
HAB2	43	6	Solitary renal cyst	43
HAB3	18	40	Recurrence HAB	58
HAB4	30	2	Solitary renal cyst	32
HAB5	30	2	Recurrence HAB	31
			Solitary renal cyst	31
HAB6	27	12	Multiple renal cysts	31
HAB7	46	14	RCC (4 cm)	47
			Solitary renal cyst	58
HAB8	41	7	Multiple renal cysts	41
HAB9	37	8	Recurrence HAB	42
HAB10	37	20	Recurrence HAB	43
HAB11	36	8	Recurrence HAB	37
HAB12	48	8	Solitary renal cyst	49
HAB13	53	4	Multiple renal cysts	55
HAB14	41	4	Multiple renal cysts	42

## Data Availability

Anonymised data available on application to the corresponding author.
